# Supplementary material for: Odd viscosity in chiral active fluids
Source: Nat Commun. 2017 Nov 17;8:1573. doi: 10.1038/s41467-017-01378-7 (PMC5691086; doi:10.1038/s41467-017-01378-7)
Supplement: Supplementary file 2 — Description of Additional Supplementary Files [file 41467_2017_1378_MOESM2_ESM.pdf]

Description of Additional Supplementary Files

File Name: Supplementary Movie 1
Description: Part 1: Relaxation of the shock profile in the case of small odd viscosity νo , leading to the steady state shown in Fig. 3a. The shock develops from a uniform state due to external forcing. We plot both the longitudinal (Burgers') shock as well as the much slower flow transverse to the shock that results from the presence of odd viscosity.

Part 2: Relaxation of the shock profile in the case of large odd viscosity ν o , leading to the steady state shown in Fig. 3c. In this case, the transverse shock develops a large amplitude and characteristic oscillations due to the interplay between odd viscosity ν o and dissipative viscosity ν
